# Probiotic Endophytes for More Sustainable Banana Production

**DOI:** 10.3390/microorganisms9091805

**Published:** 2021-08-25

**Authors:** Miguel J. Beltran-Garcia, America Martinez-Rodriguez, Ileana Olmos-Arriaga, Benjamin Valdez-Salas, Yur Y. Chavez-Castrillon, Paolo Di Mascio, James F. White

**Affiliations:** 1 Lab 309-E Building, Chemistry Department, Universidad Autonoma de Guadalajara, Zapopan 45129, Jalisco, Mexico; veki.123@hotmail.com; 2 Departamento de Biotecnologicas y Ambientales, Universidad Autonoma de Guadalajara, Zapopan 45129, Jalisco, Mexico; america.mr88@hotmail.com (A.M.-R.); ileanaolmos98@gmail.com (I.O.-A.); 3 Engineering Institute, Universidad Autónoma de Baja California, Mexicali 21280, Baja California, Mexico; benval@uabc.edu.mx; 4 Department of Biochemistry, Institute of Chemistry, University of São Paulo, São Paulo 05508-000, SP, Brazil; 5 Department of Plant Biology, Rutgers University, New Brunswick, NJ 08901, USA

**Keywords:** Banana, *Bacillus*, Cavendish, *Chryseobacterium*, Endophytic bacteria, *Enterobacter*, *Fusarium oxysporum*, Probiotics, *Pseudocercospora fijiensis*, *Pseudomonas*, Sigatoka, SynComs

## Abstract

Climatic factors and pathogenic fungi threaten global banana production. Moreover, bananas are being cultivated using excessive amendments of nitrogen and pesticides, which shift the microbial diversity in plants and soil. Advances in high-throughput sequencing (HTS) technologies and culture-dependent methods have provided valuable information about microbial diversity and functionality of plant-associated endophytic communities. Under stressful (biotic or abiotic) conditions, plants can recruit sets of microorganisms to alleviate specific potentially detrimental effects, a phenomenon known as “cry for help”. This mechanism is likely initiated in banana plants infected by *Fusarium* wilt pathogen. Recently, reports demonstrated the synergistic and cumulative effects of synthetic microbial communities (SynComs) on naturally occurring plant microbiomes. Indeed, probiotic SynComs have been shown to increase plant resilience against biotic and abiotic stresses and promote growth. This review focuses on endophytic bacterial diversity and keystone taxa of banana plants. We also discuss the prospects of creating SynComs composed of endophytic bacteria that could enhance the production and sustainability of Cavendish bananas (*Musa acuminata* AAA), the fourth most important crop for maintaining global food security.

## 1. Introduction

In 2017, bananas were ranked 12th among the top 20 commodities globally, reaching a record production of 116 million tons. Currently, around 5.2 million hectares in 135 countries are dedicated to banana production [1]. Additionally, banana is the fourth most important crop, often recognized as a staple in food security and cash crop for generating income [2]. The most common and widely exported banana is the Cavendish (AAA) group of dessert bananas (Lacatan, Robusta, Valery, Giant Cavendish, Grand Naine, dwarf Cavendish, Petit Naine, and dwarf Parfitt) that account for about 43% of global banana production [1].

Cavendish bananas are grown in nutrient-limited soils under excessive nitrogen (N) fertilization and frequently water-limited conditions [3,4]. In addition, pathogenic fungi, which are becoming increasingly virulent and resistant to fungicides, threaten banana production in major growing areas [4,5,6,7]. Banana plants have a relatively high nutrient and water demand compared to other crops; therefore, applications of high dosages of potassium (K) and N are required in banana orchards to replenish the nutrients exported from the soil to the plant and fruits [8]. It has been reported that an average of 400 Kg N ha^−1^Y^−1^ is used in the Caribbean and Latin American banana orchards, resulting in severe contamination of water bodies [9].

Due to food safety and sustainable agricultural management concerns, banana production is transitioning from conventional crop management approaches to certified and high-yielding organic systems, including microbial inoculants. However, despite the solid global trend to cultivate crops under organic management, few inoculants on the market replace chemical agents. The vast majority of commercial products are composed of *Pseudomonas* and *Bacillus* strains [10]. These observations emphasize the urgent need to develop new technologies based on plant-growth-promoting bacteria (PGPB) as microbial inoculants [11]. Interestingly, plant-beneficial bacteria living in soil as free organisms or as endophytes can trigger plant growth and protect plants from disease and abiotic factors through a wide variety of mechanisms [12,13]. Thus, promoting the propagation of these bacteria represents a potential approach for improving banana production and sustainability organically.

Endophytic bacteria reside in the internal tissues of the plant, establishing a strong symbiotic relationship that promotes plant growth and provides protection in exchange for a niche to carry out its life cycle [14]. The nature of their mutualistic association depends on their location in the plant tissue, either intercellularly or intracellularly [15,16]. Since they promote plant growth, increase crop yields, and afford disease resistance under harsh environmental conditions, endophytic bacteria are considered plant probiotics [17,18,19,20,21].

This review highlights the interest in probiotic banana endophytes as a new generation of microbial inoculants for organic banana production. We also discuss how endophytic bacterial diversity is shifted under abiotic or biotic stress conditions. Undoubtedly, understanding such changes will provide clues about which endophytic bacteria could be components of novel microbial inoculant formulas for managing specific crops. Furthermore, we describe the concept of synthetic communities (SynComs) containing keystone taxa that can support sustainable banana production.

## 2. Plant Microbiomes: The Origin of Plant Probiotic Bacteria

Plants have distinct microenvironments that harbor complex and diverse microbial communities, considered a second genome [22]. Plants have selected these microbial communities over millions of years of co-evolution to form a plant-specific microbiome, resulting in various interactions between plants and microorganisms [23]. Additionally, beneficial and detrimental microbial effects on plants can directly or indirectly affect microorganism-microorganism interactions [24].

Beneficial microbes (i.e., rhizospheric and endophytic) improve the acquisition of soil nutrients and tolerance to abiotic stresses and combat pathogens. These functions promote plant growth and consequently increase the ecological fitness of the natural environmental or agricultural system [25]. Endophytic microbes are a subset of the plant microbiome. This group of diverse and heterogeneous bacteria can easily enter plant roots through different mechanisms [26]. It is important to point out that the nature of plant-endophyte interactions can range from mutualism to pathogenicity. It was previously demonstrated that the type of interaction depends on abiotic and biotic factors, including the genotypes of plants and microbes, environmental conditions, and the dynamic networks of interactions within plant biomes [27].

Investigating the diversity and structure of a plant microbiome provides information about microbial diversity and sheds light on the function of endophytic bacteria in their plant host. Hardoim et al. [27] and Santoyo et al. [28] identified an endophytic plant microbiome composed of 21 bacterial phyla with two from Archaea. Moreover, four of the phyla, previously reported in soils and epiphytic-associated environments [29], accounted for 96% of the total endophyte microbiome. The most representative phyla of bacterial endophytes include Proteobacteria with 54% (including α, β, and γ classes), Actinobacteria (20%), Firmicutes (16%), and Bacteroidetes (6%) [27]. However, many endophytes are not culturable [30,31]. In this sense, culturable and non-culturable microbial analyses are required to comprehensively unravel the banana-endophytic community interactions [32,33,34].

More recently, microbiologists have been using high-throughput sequencing (HTS) methods to explore the structure of bacterial communities, identifying members that cannot be easily cultured in the laboratory [35,36,37], including phyla such as Planctomycetes, Verrucomicrobia, and Acidobacteria [38,39]. A significant proportion of the bacterial genera reported as endophytic is commonly found in the rhizosphere, suggesting that the endophytic microbiome may be a subpopulation of the rhizospheric bacteria [14].

It has been proposed that endophytic bacteria could be used as “plant probiotics” for microbiome reconstruction, improving crop yields, and reducing or even eliminating the requirement for chemical fertilizers [40,41,42]. Additionally, cultured bacterial endophytes display plant growth-promoting (PGP) traits, including nitrogen fixation, nutrient (e.g., nitrogen, phosphate, zinc, and other nutrient elements) and water uptake, and essential phytohormone production (e.g., indole acetic acid (IAA), cytokinin and abscisic acid) [43,44]. These bacteria indirectly provide plants with resistance or tolerance to biotic and abiotic stresses [45,46,47] by upregulating ACC deaminase activity and modulating ethylene biosynthesis [48].

Many PGP endophytic microbes are widely accepted as biofertilizers, biostimulants, and biocontrol agents [11,13,49]. These microbes exert antagonistic effects by producing antibiotic compounds, lipopeptides, cell wall degrading enzymes, volatile compounds, hydrogen cyanide (HCN), and siderophores [50,51,52,53]. Some of these beneficial microbes aggregate the soil particles to improve the soil structure and secrete extracellular metabolites to augment the breakdown of complex organic material and insoluble nutrients into simple, more available forms [54].

## 3. Synthetic Communities as Probiotic Bioinoculants

In recent years, significant steps have been taken towards understanding many facets of the plant microbiome and their interactions. With advances in sequencing technologies and analytical tools, we have learned about plant-microbial and microbial-microbial interactions and how microbes are recruited from the environment and assembled into a defined structure. These interactions are widely dependent on soil type, host genotype, and agricultural management [55]. Indeed, these types of studies have altered our perception of the complexity and dynamics of plant-microbe interactions.

Plant microbiomes have been studied by inferred functions derived from descriptive genetic data (metagenomics) and/or combined with metabolomics and culture-dependent approaches to develop synthetic microbial communities (SynComs) [56,57,58]. SynCom research and development involves employing microbial candidates as new functional probiotics for plants [57,59]. According to an analysis of 30 publications, Marin et al. [60] showed that SynComs could range from 3 to 190 microbial strains, mainly composed of bacteria belonging to the phyla Proteobacteria, Actinobacteria, Firmicutes, and Bacteriodetes.

After assembling the microbial consortium, it is then tested in plants to evaluate whether the functions and structure mimic the observed function and structure of the plant microbiome under natural conditions in the time and space of a multidimensional and complex system [55,60]. This approach reduces the complexity of the microbial community without modifying the original interactions among microbes and the host plant (Reviewed in [59,60]). A significant advantage of SynComs is that they are composed of adapting microbial communities with defined and predictable traits for crop management, producing effects that a single microbe could not generate. Additionally, due to the plasticity of SynComs in the laboratory, it is possible to understand how the plant alters its behaviors and genetic responses by removing one or several members of the consortium [60].

Plant microbiome studies are gradually considering the synergistic and cumulative effects of SynComs on different microorganisms, expanding our knowledge of plant diseases [61,62,63,64,65,66,67]. SynComs have also been designed to elucidate the specific function of plant microorganisms, including nutritional aspects (e.g., nitrogen fixation by diazotrophs) [68] and mineral assimilation (e.g., phosphate and organic nitrogen) [69,70]. Thus, the concept of SynComs for creating microbial consortia under laboratory conditions is a promising ecological strategy for developing more resilient and productive crops.

## 4. The Banana Endophytic Microbiome or Endophytome: History, Diversity, Functionality, and the Cry for Help Phenomena

According to the Scopus and Pubmed databases, as of 15 July 2021, more than 115 articles have been published on the functional properties and uses of microbial endophytes (including fungi) isolated from banana plants. We used different combinations of the search terms “banana” and “endophyte” and combinations of the following six terms: 1. Biological control, 2. *Fusarium* wilt, 3. Community diversity, 4. Plant-growth promoting traits and PGPR, 5. Endosphere, and 6. Keystone taxa. We found that biological control and *Fusarium* wilt were the most reported topics published. Many articles retrieved using these combinations were present in both databases. This observation suggests there is little information about the use of banana endophytic bacteria as probiotics or bioinoculants.

### 4.1. Pioneer Studies of Banana Endophyte

The earliest published works highlighted the antifungal, nematicide activities of reintroduced endophytic fungi from banana leaves and roots [71,72,73]. On the other hand, Esperanza Martinez-Romero and collaborators [74] published one of the first reports of endophytic diazotrophic bacterial strains, including *Enterobacter cloacae*, *Pantoea agglomerans*, *Klebsiella pneumoniae*, *K. oxytoca*, and *Rhizobium undicola*, isolated from a commercial banana plantation in Mexico. Later, Cao et al. [75] showed that the siderophore-producing *Streptomyces* could be used as a biological control agent against the causative fungus of *Fusarium* wilt disease.

Pious Thomas in India contributed substantially to research on endophytic bacteria in Cavendish bananas (Grand Naine, Dwarf cavendish). His group was among the first to combine culture- and molecular-based methods with banana tissues obtained from commercial and micropropagated material in their laboratory. He and his co-workers remarked that many bacterial endophytes remain in a viable but not cultivable (quiescent) state in micropropagated plants but can be activated after several passages (at least 20), yielding different organisms [30,31,76,77]. In addition, they proposed an approach for determining the composition of the banana microbiome, which establishes the relationships of functional diversity in the plant. In addition, their work provided exciting information about the translation of research findings from the laboratory to the agricultural field with endophytic *P. aeruginosa* from banana plants. Finally, the authors highlighted the necessity of several studies to ensure the feasibility of introducing endophytes as functional inoculants [78].

Since those pioneering studies, the search for endophytic bacteria and fungi in bananas that can be cultured for biological solutions to control crop diseases, especially those caused by fungus, has intensified. The soil-borne *Fusarium oxysporum* f. sp. *cubense* race 4 causes Panama disease [6], and *Pseudocerospora fijiensis* (previously *Mycosphaerella fijiensis*) is responsible for black Sigatoka disease [79]. Both fungal diseases are considered limiting factors for banana production worldwide.

### 4.2. The Banana Bacterial Endophytome

Recently, Nakeeran et al. [80] and Cabanás et al. [33] coined the term “banana endophytome.” That term includes all inhabiting endophytic microorganisms with a potential role as a biostimulant or biocontrol agent against pests in banana plants. Herein, we will focus on studies that have evaluated endophyte bacterial diversity in the tissues of different banana varieties. We intend to use this information to create a synopsis of the diversity and functionality of bacteria and their phyla in banana plants.

**Figure 1:**
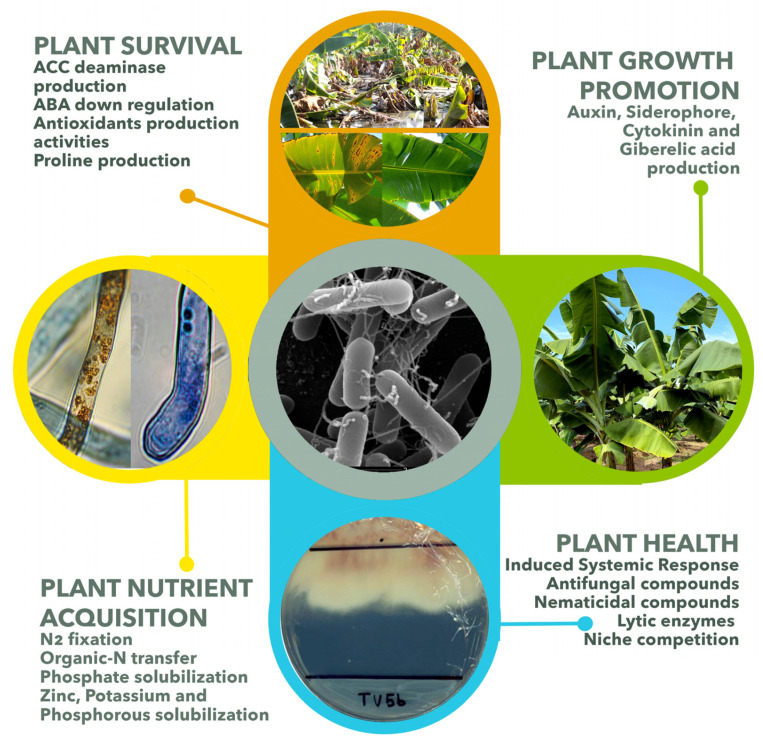
Symbiotic services/functions provided by endophytic probiotic bacteria. Endophyte bacteria can stimulate plant nutrient acquisition and health through different mechanisms and increase plant growth and production.

Figure 1 summarizes the symbiotic traits of endophytic plant bacteria. Some of their properties are widely recognized in bacteria isolated from the banana endosphere. These traits include producing growth-regulating phytohormones and acquiring nutrients for plant growth. In addition, the response to biotic stress by inducing defense mechanisms produces antifungal and nematocidal compounds and supports tolerance to various types of abiotic stress.

The endophytic bacteria of banana plants have been grouped into four major phyla: Proteobacteria, Firmicutes, Actinobacteria, Bacteroidetes, and other minor phyla such as Cyanobacteria, Chloroflexi, Verrucomicrobia, Planctomycetes, Acidobacteria, and Spirochaetes have been reported [31,33,81]. The γ-Proteobacteria is the most diverse and dominant. The most commonly isolated bacterial genera from Cavendish banana include *Bacillus* (Firmicutes), *Pseudomonas* (γ- Proteobacteria), *Klebsiella* (γ- Proteobacteria), *Enterobacter* (γ- Proteobacteria), *Rhizobium* (α-Proteobacteria), *Staphylococcus* (Firmicutes). Cumulatively, the evidence indicates that *Bacillus* and *Pseudomonas* are the most predominant genera in banana plants.

The diversity of endophytic bacterial in banana plants varies depending on the cultivar and the climatic, soil, and stress conditions. Moreover, the methods employed to quantitate these populations can influence the results and subsequent interpretation(s). For example, Rossmann et al. [82], using single-stranded conformational polymorphism (SSCP) and quantitative PCR (qPCR), reported that the pseudostem is an extraordinary microhabitat with the highest counts of endophytic bacteria (109 16SrRNA CFU gfw-1) in *Musa* sp., strain AAA EAHB (The East African Highland Cultivar group) in Uganda. Members of Enterobacteriaceae (γ-Proteobacteria) were identified as significant components in the bacterial community. The authors found that *Enterobacter* (44.6%) was predominant in plant-associated habitats, and *Pantoea* was predominant in soil (23.5%). However, in the endosphere, these values differed compared to the rhizosphere and soil. As endophytes, *Enterobacter* (31.9%), *Pantoea* (14%), *Raoultella* (12.3%), *Klebsiella* (11.4%), and *Serratia* (11.35) are considered important genera for colonizing the pseudostem. However, the most isolated genus was *Pseudomonas*. Additionally, molecular fingerprinting analyses identified dominant bands associated with *Flavobacteria*, *Sphingobacteria*, *Brevundimonas*, *Delftia*, *Herbaspirillum*, *Azoarcus*, *Acidovorax,* and *Diaphorobacter*. Undoubtedly, the predominance of Enterobacteriaceae in the endosphere is due to fertilization with animal manure in these fields. It has been reported that bananas and other plants recruit this class of bacteria from the soil for its nitrogen-fixing and antifungal properties [18,43,63,65]. On the other hand, the high presence of *Pseudomonas* is likely due to the crop proximity to *Fusarium* wilt-infested fields. In Uganda, this disease has been incredibly destructive for banana plants.

In another study conducted in Africa (Kenya), Ngamau et al. [83] isolated and identified endophytic bacteria from *Musa* AAA-Cavendish and *Musa* AAB plantain bananas. The authors selected 214 isolates and grouped them into one of three families: Bacillaceae, Pseudomonadaceae, and Enterobacteriaceae. According to the 16S rRNA gene results, the Enterobacteriaceae family was the most diverse with eight genera: *Serratia*, *Rahnella*, *Enterobacter*, *Yokenella*, *Raoultella*, *Klebsiella*, *Yersinia,* and *Ewingella*. Both the Pseudomonadaceae and Bacillaceae families were represented by only one genus *Pseudomonas* and *Bacillus*. Interestingly, 100% of the bacterial isolates could fix nitrogen, 62% exhibited phosphate solubilization activity, and 12 *Pseudomonas* isolates displayed siderophore production. Notably, that microbial community matched with the ongoing decline in soil fertility in Kenya. However, these results contrast with those obtained by Brazilians evaluating bacterial populations in Prata Anã (AAB) bananas.

Souza et al. [84] and Andrade et al. [85] reported that the predominant phyla in Prata Anã (AAB) roots consisted of 70% Firmicutes and 30% Proteobacteria. Further analyses revealed that the isolates were from 15 species belonging to ten genera: *Agrobacterium*, *Aneurinibacillus*, *Bacillus*, *Enterobacter*, *Klebsiella, Lysinibacillus, Micrococcus*, *Paenibacillus*, *Rhizobium,* and *Sporolactobacillus*. The genus *Bacillus* was the most frequently identified (87.3%), followed by *Lysinibacillus* (3.9%).

Pereira et al. [86], from the same research group in Brazil, characterized 39 bacterial isolates from roots and grouped them into 4 genera: *Bacillus*, *Rhizobium*, *Klebsiella,* and *Enterobacter*. The genus *Bacillus* occurred more frequently (92.5%) than the other genera, which only contained one representative of each genus (2–5%). The identified *Bacillus* species included: *B. subtilis* (as the most predominant species), *B. pumilus*, *B. safensis*, *B. altitudinis*, *B. thuringiensis*, *B. cereus*, *B. amyloliquefaciens*, *B. axarquienses,* and *B. megaterium*. None of the identified isolates, including the *Rhizobium* strain, could fix atmospheric nitrogen.

The cultivar Prata Anã is grown in a semi-arid environment in Brazil and has a narrow association with non-diazotrophic bacteria. However, 90% of the bacterial isolates were capable of reducing nitrate to nitrite. Nitrate reductase catalyzes the first step in the reduction of nitrate to ammonium for the N-organic synthesis. In addition, only 30% of the isolates were verified as urease positive, including *Bacillus*, *Klebsiella* and *Enterobacter* [86]. It is well-known that urease catalyzes the hydrolysis of urea to ammonium and CO_2_. Although the bacteria are non-diazotrophic, the enzymatic ammonium production and its subsequent transformation to organic nitrogen are sufficient for maintaining plant growth under nitrogen-deficient conditions, as demonstrated in other plant-endophyte systems [17]. Therefore, the abundance of Firmicutes in the Prata Anã roots with these enzymatic capabilities is strategic to survive naturally in a semi-arid environment and determines the use of mineral nitrates as fertilizer.

Using culture-dependent and culture-independent methods, Thomas and Sekhar [81] revealed differences in bacterial diversity of typically unculturable bacteria prevailing in sucker shoot-tips of Grand Naine cultivar (cv.) bananas compared to the culturable bacteria. In that study, the cultivable bacteria included 37 strains, including 16 genera and 24 species distributed almost equally among three phyla: Actinobacteria (36.1%), Proteobacteria (33.3%) and Firmicutes (30.6%). *Klebsiella pneumoniae,* and *K. oxytoca* were the most common species.

Furthermore, a metagenomic method reported enormous bacterial diversity in banana plants. In one study, Proteobacteria was the dominant group (64%), followed by Firmicutes (12.1%), Actinobacteria (9.5%), Bacteroidetes (6.4%), Planctomycetes and Cyanobacteria and others (>1%) contributing 14 phyla such as Acidobacteria and Verrucomicrobia and the domain Euryarcheota. Class distribution of the OTU (%) values showed that the bacterial community consisted of γ-Proteobacteria (42.6%), α-Proteobacteria (14%), Actinobacteria (9.01%), Clostridia (6.5%), β-Proteobacteria (6%), and Bacilli (5.6%) [81]. It is important to point out that the γ-Proteobacteria included agriculturally important genera such as *Acinetobacter*, *Acetobacter*, *Klebsiella*, *Pseudomonas*, *Stenotrophomonas* and *Serratia*.

Marcano et al. [87] published a study on endophytic bacteria associated with the roots of Cavendish banana plants under organic management in the Dominican Republic. They highlighted the presence of a *Pseudomonas plecoglossicida* strain that improved fruit yield and controlled black Sigatoka outbreaks. The 114 isolates belonged to 20 different genera, predominantly *Bacillus*, *Pseudomonas*, *Enterobacter,* and *Stenotrophomonas*. Bacteria from the genera *Acinetobacter*, *Pantoea*, *Citrobacter*, *Lysinibacillus*, *Pseudoxanthomonas*, *Comamonas,* and *Rhizobium* and others were considered minor genera. At the phylum level, it was found that banana roots contain 63% Proteobacteria (α-, β- and γ-Proteobacteria) and 37% Firmicutes (only *Bacillus* and *Lysinibacillus*). The application of some of these endophytes was shown to reduce black Sigatoka disease severity in organic plantations in Colombia.

Cultivated endophytic bacteria isolated from leaves of a red banana variety (M. acuminata AAA, red Dacca) were grouped into three phyla: Actinobacteria, Proteobacteria, and Firmicutes, comprising 8 genera and 10 species. Most of the isolates belonged to Firmicutes (40.7%), followed by Proteobacteria (41.11%) and Actinobacteria (11.76%). Moreover, the dominant bacterial genera in the red banana included *Bacillus* (36.97%) and *Klebsiella* (29.41%) [88].

Cabanás et al. [33] studied the structure, composition, and co-occurrence relationships of dwarf Cavendish banana root endophytome in mother plants and suckers in banana plantations in the Canary Islands. They collected >1000 culturable root endophytes. Culturable and non-culturable (i.e., HTS) approaches have indicated low microbial diversity within the banana root endosphere. According to their metagenomic results, Proteobacteria was the predominant phylum (72.3%), followed by Actinobacteria (12.1%) and Firmicutes (1.3%). Furthermore, based on the co-occurrence network analyses, *Pseudomonas* was the dominant genus, playing a vital role in the endophytic root microbiome.

The dwarf Cavendish banana plant root endosphere core bacteriome was shown to be composed of few genera, with 41% of the total sequences distributed among *Pseudomonas* (27%), *Rhizobium* (8%), and *Streptomyces* (6%). Proteobacteria, Bacteroidetes, and Actinobacteria were among the most dominant cultivable bacteria. Additionally, the genus *Enterobacter* was the fifth most abundant.

**Figure 2:**
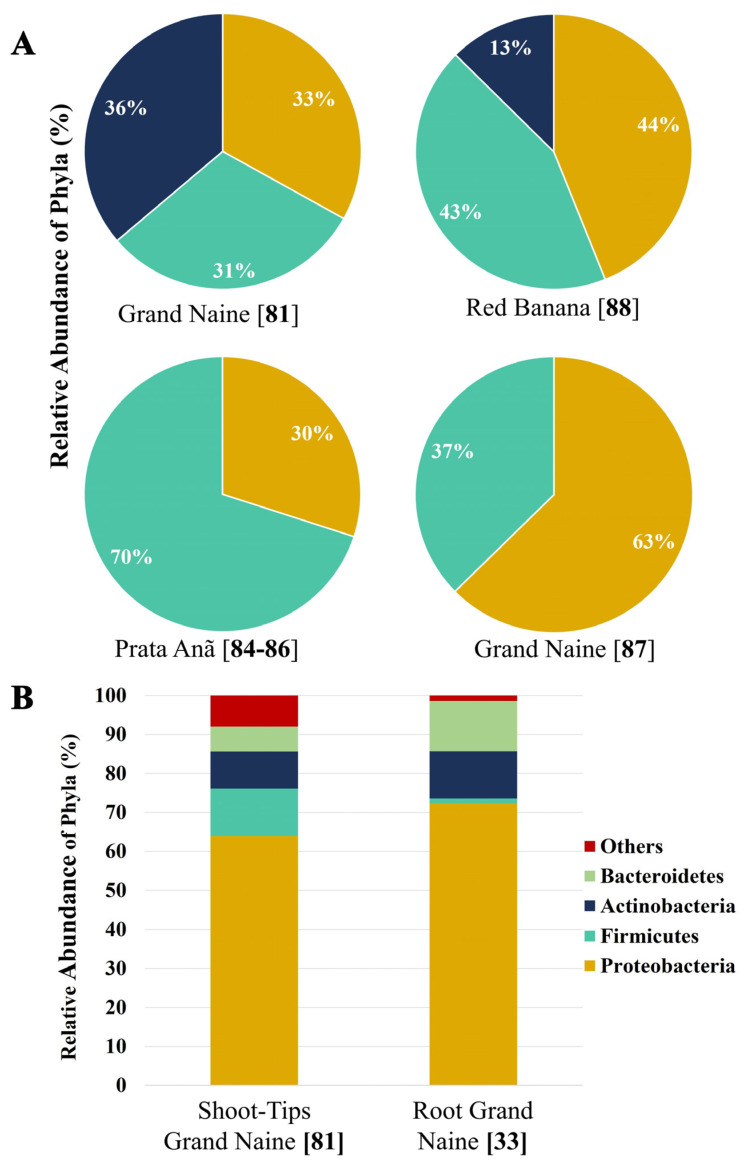
Relative abundance of cultivable and non-cultivable endophytic bacteria at phylum level from different cultivars of banana. (**A**) Comparative analysis among cultivable endophyte populations of shoot tips [81], roots under organic management [87], and red banana leaves of banana Cavendish AAA [87] and the Grand Naine under organic management. In the three tissues, the Proteobacteria was the predominant phylum. Leaves lacked members of the Actinobacteria phylum. Interestingly, Prata Anã recruits more Firmicutes to the roots. (**B**) Non-cultivable analysis of root endophytes. Both studies showed that Proteobacteria is the main microbial phylum in Cavendish bananas. The averages were calculated using data from previously published studies [33,81,84,85,86,87,88].

These studies demonstrate that the method of analysis, cultivar, and microhabitat (root, pseudostem, and leaf) are influential factors for endophytic bacteria diversity in banana plants. Figure 2 shows the relative abundance of endophytic bacteria that have been reported in some studies mentioned in this review. The studies were carried out in different tissues of banana plants (Dwarf Cavendish and red banana AAA) and Prata Anã (AAB) varieties grown in commercial plantations exposed to different stress factors (2A). We can also observe the influence of the analysis method (cultivated and non-cultivated) on the distribution of the endophytic microbial community (2B).

It is important to note that all published works about the diversity of banana endophytes have contributed to the knowledge of bacterial diversity and will help discover endophytic bacteria that could be candidate members of probiotic SymComs for banana cultivation. Additionally, our literature review revealed that combining culturable and non-culturable approaches facilitates the visualization of essential plant microorganisms or “keystone taxa.” Keystone species play a significant role in shaping functional microbial networks, and their loss compromises the microbiome stability and the services they offer to plants [89,90]. Next-generation sequencing (NGS) studies have suggested that complex plant-microbe networks cope efficiently with environmental stresses, including those caused by pathogens. As we will discuss later, and based on the functionality of endophytic strains, various authors have suggested that *Pseudomonas*, *Klebsiella*, *Enterobacter*, *Paenibacillus*, *Sphingophix*, *Micrococcus,* and *Rhizobium* are keystone bacteria for banana plants [18,33,91]. For more information about keystone taxa, we direct the reader to the following publications [92,93,94,95].

### 4.3. Fusarium Wilt Disease Shifts Endophytic Communities in Banana Plants

Before introducing how biotic stress caused by *Fusarium* wilt disease alters the diversity of endophytic bacteria in bananas, it is important to note that plants under biotic and abiotic stress elicit a “cry for help” response mechanism to recruit both beneficial soil and rhizosphere microorganisms [96]. Plants adjusting their root exudate composition, mainly organic acids such as malate and oxalate, can replace inorganic phosphate (P_i_) bound in insoluble P_i_-complexes via metal ion chelation or anion exchange increasing root P uptake [97]. In addition, when attacked by pathogens, plants activate and assemble protective microbiomes, which help the plants resist and withstand diseases.

Previously, Rudrapa et al. [98] showed that malate efflux induces *B. subtilis* attraction to *Arabidopsis* roots during *Pseudomonas syringae* infection. The attracted *B. subtilis* triggered systemic resistance and protected plants against *P. syringae*. The “cry for help” concept was recently supported by a field experiment in which durum wheat naturally infected by *Fusarium graminearum* was enriched with *Stenotrophomonas rhizophila* in the rhizospheres and root endospheres to alleviate fungal disease [99]. This protection mechanism has not been described in banana plants. However, evidence shows that the microbial community shifts in banana plants infected by *Fusarium*, indicating a “cry for help” mechanism.

For example, *Fusarium* wilt disease has been reported to induce drastic alterations in the microbial endophyte diversity, especially γ-Proteobacteria and Bacteroidetes populations, in Cavendish bananas [91,100]. Lu et al. [101] observed attenuated Proteobacteria abundance in the roots, stems, and leaves from banana plants in a disease conducive orchard compared to the levels in a disease suppressive orchard. These authors also observed a 45% increase in Bacteroidetes abundance in the roots of plants in the disease suppressive orchard. Interestingly, *Chryseobacterium*, which belongs to the Bacteroidetes phylum, replaces these Proteobacteria strains. The increased abundance of *Chryseobacterium* as an endophyte or rhizosphere inhabitant has been related to its demonstrated antifungal protection and phosphate solubilization in various crops. In addition, plant protection against fungal pathogens is augmented when *Chryseobacterium* is placed in a network with *Enterobacter*, *Pseudomonas*, *Bacillus,* and *Stenotrophomonas* [102,103,104,105].

Lu et al. [101] also reported that the main change observed at the genus level in samples collected from the *Fusarium* wilt suppressive plantation was the abundance of *Pseudomonas* (γ-Proteobacteria). Several *Pseudomonas* strains antagonistic to FocTR4 were collected from the disease suppressive orchards, with *P. putida*, *P. aeruginosa*, *P. fluorescens*, *P. lutea,* and *P. monteilii* being the most antagonistic. An increase in *Sphingomonas* (α-Proteobacteria), *Agromyces* (Actinobacteria), and *Rhizobium* (α-Proteobacteria) and a 7-fold reduction in *Bacillus* were also observed in the suppressive orchards. This observation highlights the plant’s urgency to acquire rapidly growing Proteobacteria that can transfer nutrients (N and P) to the plant and synthesize ACC deaminase and IAA auxin.

Liu et al. [91] detected a shift in the Enterobacteriaceae family during *Fusarium* wilt infection. The relative abundance of endospheric bacteria belonging to the Proteobacteria, Firmicutes and Actinobacteria phylum was positively associated with disease suppression, leading the authors to propose *Klebsiella* spp. as a keystone bacterium. Indeed, *Pseudomonas*, *Enterobacter*, and *Klebsiella* promote banana plant growth by modulating nitrogen fixation, phosphate solubilization, IAA production, and antifungal agents in plant tissues [106,107,108].

Additionally, Köberl et al. [100,109] showed that healthy banana plants collected in *Fusarium* wilt disease fields contained increased *Pseudomonas* and *Stenotrophomonas* abundance. It is plausible that these microorganisms could be health indicator markers. These γ-Proteobacteria were also found to increase in the microbiome of Gros Michel bananas grown under agroforestry conditions using Inga trees (green manure) for nitrogen fertilization [110].

Beyond the cultivar, geographic location, and fertilization management, it is consistent that a shift of banana endospheric microbial communities structure is consistently observed [80,89]. However, to understand the “cry for help” mechanism and keystone candidates relationship, we must combine biological and chemical approaches [111], especially when designing SynComs that promote resilience to all types of stresses. Despite the progress made, we still need to know if the observed changes of γ-Proteobacteria abundance in *Fusarium*-infected plants occur in other diseases such as black Sigatoka.

### 4.4. Banana Endophyte Probiotics for Black Sigatoka

In addition to *Fusarium* wilt or Panama disease, another fungus, *Pseudocercospora fijiensis*, threatens global banana production. This pathogen causes banana leaf necrosis, reducing their photosynthetic capacity, influencing the filling of the fruit, inducing premature ripening of fruit harvested, and consequently reducing the economic gain to zero [79].

In the 1960s, the appearance of the black Sigatoka disease completely modified the agricultural management of banana crops. An extensive application of chemical fungicides was used as a first option to combat this disease. However, this strategy quickly became ineffective due to the emergence of resistant and tolerant *P. fijiensis* populations to the chemical agents (e.g., carbendazim, azoxystrobin, propiconazole and mancozeb) [5,112]. Another strategy involved removing the leaves with lesions weekly (at the first streak stage) to reduce the amount of inoculum (ascospores). However, this practice reduced the number of functional leaves needed to maintain fruit quality once the banana bunch formed.

Once the *P. fijiensis* mycelium penetrates the stomata, after six days of epiphytic growth, it colonizes intercellular spaces and maintains a biotrophically relationship with the plant, behaving such as an endophyte. However, the plant’s responses, including the production of hydrogen peroxide (H_2_O_2_) and the fungal secretion of melanin pigment into the foliar tissue, modify the behavior of the fungus, transitioning from a biotrophic to a necrotrophic stage [113]. Therefore, the use of endophytic bacteria to reduce the impact of black Sigatoka appears to be a viable option.

Given the demand of the consumer market and the need for clean, sustainable agriculture, the world’s banana production is shifting from conventional management practices towards certified organic practices. Biological products developed for managing black Sigatoka include plant extracts, *Bacillus* and *Trichoderma* spores, selected for their antifungal activity, and *Saccharomyces cerevisiae* (Agro-Mos) cell extracts [114]. However, the environmental conditions limit the biological control agent due to its low survival on the leaves or in the soil [78].

A few scientific works have reported the potential use of endophytes as biological control agents against black Sigatoka in both the laboratory and banana fields. In the Dominican Republic, Marcano et al. [115] showed the potential of endophytic *Bacillus* strains isolated from roots of Dwarf Cavendish against Sigatoka pathogen in vitro and in growth chamber assays. The best fungus controllers were *B. licheniformis*, *B. siamensis*, *B. subtilis* ssp. *Inaquosorum,* and *Rhizobium massiliae*. Notably, plants co-inoculated with bacteria and the pathogen displayed attenuated disease severity indices, an effect assigned to an induced systemic response (ISR) phenomenon.

In another study, previously mentioned in this review [87], a probiotic formulation of endophyte bacterial strains isolated from banana roots under organic management was developed. After a strict selection based on PGP-properties, two strains of *Pseudomonas* (*P. plecoglossicida* and *P. taiwanensis*) were found to retard foliar necrosis symptoms in field trials. The control plants presented necrosis symptoms from the first leaf (i.e., the youngest leaf). In contrast, plants treated with endophytic bacteria only showed necrosis symptoms at the fourth leaf (i.e., older). Furthermore, the fruit and the bunch’s average weight was higher in banana plants treated with the endophyte probiotic bacteria.

Our research group has studied black Sigatoka from two scientific approaches to reduce the impact of black Sigatoka in bananas: a) the biochemistry and physiology of *P. fijiensis* and b) the endophytic bacteria populations and their usefulness as probiotics in commercial plantations of Colima and Jalisco, Mexico [5,18,116,117,118]. First, we proposed *E. cloacae*, which is widely distributed in banana plants and seeds [18,30,119,120], as a keystone member of the microbial community of these plants. This endophyte serves as a plant-growth promoter, contributes to banana plant nutrition and imparts fungal disease tolerance [33,80,100,110].

The *E. cloacae* (C2) strain studied in the laboratory (GenBank access KU93327) is a diazotrophic endophyte producer of siderophores and auxins with low ACC deaminase activity. This bacterium can inhibit different fungicide-resistant strains of *P. fijiensis* in vitro. Inoculation with the C2 strain has been shown to stimulate plant growth in the absence of nutrients, especially nitrogen. In this situation, plants ‘consume’ soil bacteria to obtain nutrients [11,17,26,121]. In this process, termed ‘rhizophagy’ or the ‘rhizophagy cycle’, soil bacteria are attracted to roots by root exudates, internalized into root cells at the root tips and subjected to superoxide secreted by root cells to extract nutrients from the internalized bacteria. Any surviving bacteria may be ejected back into soils from tips of root hairs thus that they can acquire additional nutrients [11,18,26]. Interestingly, an extended nutrient-transfer symbiosis is established in bananas, where *E. cloacae* appear to stably function in the rhizophagy cycle in banana roots [18]. The mechanism was verified by tracking ^15^N in pheophytin (a molecule derived from plant chlorophylls) after inoculation with ^15^N-labeled *E. cloacae*. The relative abundances of pheophytin isotopomers indicate ^15^N label incorporation into three of the four nitrogen atoms of tetrapyrrole, confirming the N-transference from bacterium to plant tissues. Moreover, *E. cloacae* continued to be internalized into the banana roots after 60 days, a process that can be accelerated for up to 48 h in roots pretreated with silver nanoparticles (Macedo-Raygoza, personal communication).

A synthetic probiotic formula for the treatment of black Sigatoka has been developed. This microbial formula includes bacterial endophytic strains collected between 2009-2011 from functional leaves without apparent symptoms of black Sigatoka, including *E. cloacae* (C2), *Bacillus velezensis* (GenBank access. MT 919309), *B. subtilis* (Genbank access. MT919310) and *Lysinibacillus sphaericus* (Genbank access. MW486577) strains. This probiotic formula reduced the impact of fungal disease on naturally infected plantations in Colima, Mexico. After one year of applying this product, growers eliminated the requirement of administering chemical fungicides (e.g., mancozeb and propiconazole, typically applied every 5 and 15 days, respectively). Additionally, when the plantation transitioned to organic management, the applications of commercial plant extracts (e.g., Banacore^®^, Biotika Gober^®^ and Timorex Gold^®^) were reduced by 40–50%. The probiotic inoculant is currently patent pending (PCT/Mx2021/000006 and MX/a/2021/002192) [119].

## 5. Conclusions

The use of plant probiotics can prevent plant disease, increase agriculture production, attenuate chemical inputs, and reduce greenhouse gas emissions, resulting in more sustainable agricultural practices. While the application of commercial bioinoculants is a good strategy for minimizing the use of agrochemicals, these products should only be employed after careful considerations. Indeed, the effects of applying large quantities of commercial *Bacillus* strains or other microbes on the native soil and endosphere microbial structures are still not fully understood.

However, microbial endophytes (bacteria and fungi) have been shown to benefit the host plants directly or indirectly by producing plant-promoting traits and helping to defend the plant against pathogens. Herein, we presented a substantial amount of information about the widely conserved endophytic bacterial communities and their potential roles as probiotics in Cavendish banana plants. However, there are challenges in applying the products to commercial plantations. Based on HTS and cultured-based methods, we also provided evidence that supports *Chryseobacterium*, *Enterobacter*, *Klebsiella*, *Micrococcus*, *Paenibacillus*, *Pseudomonas Rhizobium,* and *Sphingophix* as keystone taxa for banana plants.

Furthermore, under biotic stress conditions, especially Panama disease, shifts in banana endophyte communities have been reported. These observations provide valuable insights that can be used for developing bioinoculants composed of native endophytic bacteria that could be evaluated in field studies. SynComs are an attractive approach for alternative agricultural management strategies. Undoubtedly, endophytic probiotics represent a novel pathway for improving banana crop performance, especially in terms of enhancing banana resiliency to environmental stresses and fungal diseases such as *Fusarium* wilt and black Sigatoka disease
